# Effect of Plastic Composition in the Combustion Material on the Persistent Organic Pollutant Content in Smoked Dried Shiitake Mushroom

**DOI:** 10.1155/jamc/1257545

**Published:** 2026-08-04

**Authors:** Uyen Nguyen-Thu, Giang Do-Hoang, Luyen Nguyen-Thi, Minh Nguyen-Thi-Thu, Duong Hoang-Thuy, Le Bui-Thi-Nhat, Ngoan Nguyen-Thi, Anh Hoang-Le-Tuan, Tung Nguyen-Ngoc, Dat Nguyen-Tien

**Affiliations:** ^1^ High Technology Innovation Center, Vietnam Academy of Science and Technology, 18-Hoang Quoc Viet Nghia Do, Hanoi, Vietnam, vast.ac.vn; ^2^ University of Science and Technology of Hanoi, Vietnam Academy of Science and Technology, 18-Hoang Quoc Viet Nghia Do, Hanoi, Vietnam, vast.ac.vn

**Keywords:** PAHs, PCBs, PCDD/Fs, plastic contamination, POPs, shiitake mushroom, smoking

## Abstract

Smoking is a traditional preservation method in Vietnam, widely applied to shiitake mushrooms (*Lentinula edodes*) as well as meats, yet the use of plastic‐contaminated fuels poses emerging food safety concerns. This study provides the first congener‐specific characterization of polycyclic aromatic hydrocarbons (PAHs), polychlorinated biphenyls (PCBs), and polychlorinated dibenzo‐*p*‐dioxins and furans (PCDD/Fs) in smoke‐dried shiitake produced with wood and wood adulterated by polyethylene (PE), polystyrene (PS), or polyvinyl chloride (PVC). Using GC–MS/MS combined with multivariate analyses, we observed clear differences in contaminant accumulation driven by fuel composition. Clean wood (W) yielded the lowest burdens, with PCB levels < 150 ng/kg, PCDD/Fs negligible, and PAHs < 650 µg/kg. Plastic‐adulterated fuels significantly increased contamination (Kruskal–Wallis *p* < 0.001), with PAH and PCB/PCDD/F levels following robust gradients of W < PE < PS < PVC (PCBs/PCDD/Fs) and W < PE < PVC < PS (PAHs). PE was characterized by light PAHs (naphthalene, acenaphthylene, and acenaphthene) and moderate PCB increases; PS, by high‐molecular‐weight PAHs and coplanar PCBs; and PVC, by the broadest enrichment, including nearly all tested PCDD/F congeners. Hierarchical cluster analysis and PCA confirmed clustering by fuel type and revealed pollutant fingerprints specific to each plastic. These findings demonstrate, for the first time, the toxicological risks of plastic‐contaminated smoking fuels in Vietnamese shiitake processing and provide baseline data essential for risk assessment and food safety policy.

## 1. Introduction

Smoking has long been a traditional method of food preservation, valued for its ability to extend shelf life and impart distinctive sensory qualities. The process relies on the combustion of natural fuels such as wood or straw, generating smoke rich in antioxidant, antimicrobial, and flavor‐enhancing compounds [1]. In Vietnam, smoking is not only applied to meats such as buffalo or pork but is also widely practiced for preserving shiitake mushrooms (*Lentinula edodes*), which are an important dietary component and export commodity. Smoke‐drying remains one of the most common preservation methods for shiitake mushrooms, allowing mushrooms to be stored for extended periods without refrigeration while retaining their characteristic aroma and texture.

Despite its cultural and economic importance, the use of nontraditional fuel sources in smoke‐drying poses emerging food safety risks. In many rural settings, plastic‐containing packaging waste or painted and treated woods are inadvertently burned together with firewood. The incomplete combustion of plastics, especially chlorine‐containing polymers (e.g., polyvinyl chloride [PVC]), can release a wide spectrum of toxicants, including polycyclic aromatic hydrocarbons (PAHs) and dioxin‐related compounds (DRCs) such as polychlorinated dibenzo‐p‐dioxins, dibenzofurans, and dioxin‐like PCBs [2, 3]. These pollutants are of major concern because of their persistence, lipophilicity, and toxicological properties. Dioxins, furans, and PCBs are classified by the World Health Organization as human carcinogens [1, 3–5] and have been associated with endocrine disruption, immune suppression, developmental toxicity, and elevated risks of cancers and chronic diseases [6–10]. Due to the toxicological risks associated with these contaminants, several international regulations, including European Union legislation, have established maximum limits for PAHs and DRCs in smoked food products [11].

Although several studies worldwide have documented contamination of smoked foods with DRCs and PAHs, no studies have specifically reported the congener‐specific profiles of PAHs, PCBs, and dibenzo‐p‐dioxins and dibenzofurans (PCDD/Fs) in traditionally smoke‐dried shiitake mushrooms in Vietnam. Based on searches of the available literature related to smoked shiitake mushrooms, PAHs, PCBs, PCDD/Fs, and plastic‐contaminated fuels, no directly comparable studies were identified. Therefore, this study provides, to the best of our knowledge, the first characterization of these contaminant profiles under such processing conditions. By combining targeted chemical analysis with multivariate statistics, this work not only demonstrates the substantial increase in toxic contaminants linked to plastic‐adulterated fuels but also provides a novel chemical fingerprint of how different plastics (polyethylene [PE], polystyrene [PS], and PVC) alter pollutant burdens in a widely consumed food. These findings highlight the urgent need for controlling fuel quality in traditional food processing and contribute essential baseline data for risk assessment and food safety regulation in Vietnam.

## 2. Materials and Methods

### 2.1. Samples

Nine fresh shiitake samples were collected from local markets, thoroughly washed with water, and allowed to drain. The samples were then divided into five equal parts. These were subsequently smoked using different smoking materials: pure wood without impurities (Group W) and wood mixed with one of the following plastics: polyethylene (PE), polystyrene (PS), and polyvinyl chloride (PVC) pellets, at the ratio of 99/1 (w/w). The wood used in all treatments was *Manglietia conifera* obtained from local commercial firewood sources. Before use, the wood was thoroughly washed and air‐dried. The smoking process was conducted in a custom‐built box‐type smoking chamber equipped with a separate combustion compartment and airflow control system. The distance between the combustion source and the sample chamber was maintained at approximately 120 cm to avoid direct flame contact. The smoking process was carried out for five consecutive days, with 6 h of smoking per day, while the chamber temperature was maintained within the range of 80°C–90°C. To minimize cross‐contamination, each fuel type was combusted in separate smoking sessions. The fifth portion of each sample was oven‐dried at 90°C until a constant weight was obtained. Following treatment, all samples were freeze‐dried, ground into fine powder, and stored at −20°C until further analysis of their chemical constituents.

### 2.2. Experimental

The samples were processed and analyzed following the same procedure as described in our previous publication [12, 13]. Briefly, the samples were freeze‐dried, the fat content was determined, and persistent organic pollutants (POPs) were extracted and enriched, followed by analysis using a GC–MS/MS system. Data were processed and extracted with XCalibur 4.2 software (Thermo Scientific, USA), and subsequent statistical analyses were performed using R software (R Foundation for Statistical Computing, Vienna, Austria). A detailed description of the procedure is provided in the supporting section.

## 3. Results

### 3.1. PCDD/F and PCB Contents in the Samples

PCBs are a class of chlorinated biphenyls that are recognized as persistent, bioaccumulative, and carcinogenic pollutants [14]. Their toxicity is strongly influenced by the position and number of chlorine substitutions, which allows congeners to be classified into coplanar (nonortho) and noncoplanar categories. Coplanar congeners adopt an almost planar geometry that closely resembles the structure of polychlorinated PCDD/Fs, and for this reason, they are designated as dioxin‐like PCBs (DL‐PCBs) [15]. Because both DL‐PCBs and PCDD/Fs are ligands of the aryl hydrocarbon receptor (AHR), though with distinct binding strengths and toxic potencies, coplanar and mono‐ortho PCBs are regarded as contributors to dioxin‐like toxicity and are evaluated together with PCDD/Fs under the World Health Organization toxic equivalency factor/toxic equivalency (WHO TEF/TEQ) scheme [6, 16]. Two benzene rings characterize PCDD/Fs themselves joined through oxygen bridges: in dioxins (PCDDs), two oxygens connect the rings, whereas in furans (PCDFs), a single oxygen forms the bridge; in both cases, one to eight chlorine atoms may be substituted, producing multiple families of congeners with varying characteristics [4, 17–19].

The contents of PCDD/Fs and PCBs in smoked shiitake samples are presented in Table S1. The analysis revealed clear differences in contaminant accumulation among shiitake mushrooms smoked with different fuel materials. Samples smoked with clean wood (W) exhibited the lowest PCB levels, generally below 150 ng/kg, and negligible PCDD/Fs. In contrast, mixtures of woods with plastics markedly increased contamination. Mushrooms smoked with woods and PE (PE group) showed intermediate PCB levels around 480–520 ng/kg and PCDD/Fs below 1 ng/kg, while those with woods and PS (PS group) had higher burdens, averaging 680–750 ng/kg PCBs and approximately 3 ng/kg PCDD/Fs. The PVC group (smoked by woods and PVC) displayed the highest contamination, exceeding 1000 ng/kg for PCBs and reaching above 9 ng/kg for PCDD/Fs, indicating a substantial release of POPs. Statistical analysis corroborated these observations. A Kruskal–Wallis test demonstrated highly significant differences among the four groups for both PCBs (*H* = 32.84, *p* < 0.000001) and PCDD/Fs (*H* = 32.87, *p* < 0.000001). *P*ost‐hoc pairwise comparisons with Benjamini–Hochberg correction Table S2 confirmed that all group pairs differed significantly (adjusted *p* ≈ 0.0004), including W vs PE, W vs PS, W vs PVC, PE vs PS, PE vs PVC, and PS vs PVC.

**FIGURE 1:**
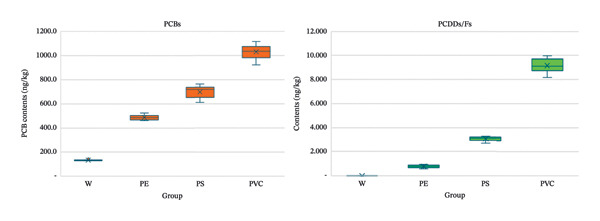
The boxplot of total PCB (left) and total PCDD/F (right) contents of the samples.

These results indicate that the increase in contamination followed a statistically robust gradient of *W* < PE < PS < PVC for both PCB and PCDD/F accumulation. The boxplot distributions (Figure 1) further visualized these trends, showing nonoverlapping interquartile ranges between the groups, particularly between W/PE and PVC. Together, the quantitative analysis and statistical testing demonstrate that contamination does not simply increase in magnitude but follows a clear and statistically validated gradient driven by the presence and type of plastic additives, with PVC contributing the highest toxic burden.

**FIGURE 2:**
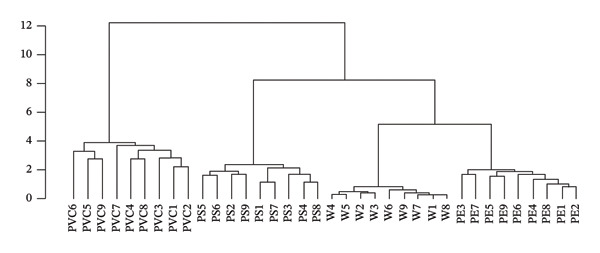
Hierarchical cluster analysis dendrogram of the PCDD/Fs and PCBs in smoked shiitake samples.

Building upon the congener profiles, the hierarchical cluster analysis (HCA) further illustrated the grouping of smoked shiitake samples according to fuel type (Figure 2). The dendrogram clearly separated the four treatment groups, with W and PE forming a closer cluster, reflecting their relatively lower contaminant loads, while PS and PVC clustered independently, consistent with their higher PCB and PCDD/F burdens. Within‐group clustering was tight, indicating good reproducibility of contamination patterns across replicates. The proximity of PE to W suggests that PE‐adulterated wood produces emissions more similar to clean wood, whereas the distinct separation of PVC highlights its unique and pronounced contribution to the generation of DRCs. These clustering results corroborate the boxplot trends and reinforce the conclusion that fuel composition exerts a dominant influence on the contamination profiles of smoked mushrooms.

**FIGURE 3:**
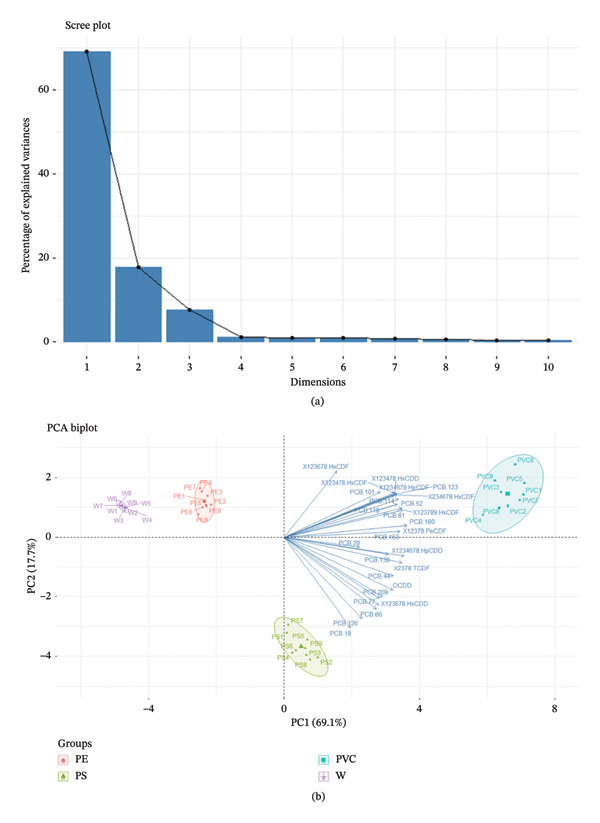
The PCA (a) scree plot and (b) biplot of PCBs and PCDD/Fs contents of the samples.

Principal component analysis (PCA) provided further insights into the separation of smoked shiitake samples according to fuel type. The scree plot indicated that the first two principal components accounted for the majority of the total variance, with PC1 and PC2 together explaining 86.8% of the variation, thus offering reliable discriminatory power. Eigenvalue analysis showed that only the first three PCs had eigenvalues greater than one (18.65, 4.78, and 2.04, respectively), supporting the use of two to three principal components for group differentiation. The loading plot (Figure S1) highlighted the contribution of individual congeners to the PCA model, enabling interpretation of pollutant signatures associated with specific fuel types. When combined with the sample distribution in the PCA biplot (Figure 3), the results revealed clear pollutant patterns. Shiitake smoked with clean wood (W) showed PCB contamination but virtually no PCDD/Fs, and statistical comparisons confirmed that W samples differed significantly from all other groups for every compound tested (adjusted *p* < 0.001). In contrast, the addition of PE led to notable increases in congeners such as PCB 28, 12378‐PeCDF, and 123678‐HxCDF. Post‐hoc tests indicated that while PE was significantly different from W (*p* < 0.001), its levels of PCB 28 and PCB 153 were not significantly different from those in PS or PVC (*p* > 0.05), suggesting an intermediate position. Stronger PCB accumulation was observed when PS was present, with marked elevations in PCBs 18, 66, 77, 126, and 209, along with dioxin congeners including 123678‐HxCDD and OCDD; statistical analysis confirmed that PS levels of these compounds were significantly higher than in PE (*p* < 0.001). The PVC group showed the most distinct profile, characterized by broad enrichment of nearly all tested PCDD/F congeners, accompanied by elevated levels of PCBs such as PCB 101, 114, 118, 81, 153, and 180. Pairwise comparisons confirmed significant differences between PVC and all other groups for the majority of congeners (adjusted *p* < 0.001), with only a few exceptions, such as PCB 28 and 123678‐HxCDF, where PE and PVC did not differ significantly.

These results are consistent with the HCA clustering, which grouped samples by fuel type, and further illustrate how the chemical fingerprints of contaminants correspond directly to the composition of the smoking material. An additional observation is that PC1 primarily separated PVC from the other groups, reflecting the dominant influence of dioxin‐like compounds and high‐chlorinated PCBs, while PC2 distinguished PS from PE and W, driven by mid‐ to lower‐chlorinated PCBs. This distribution underscores the role of chlorine substitution patterns in shaping the contaminant profiles, thereby linking the mechanistic classification of PCBs and PCDD/Fs with their empirical accumulation in smoked shiitake mushrooms.

### 3.2. PAH Contents in the Samples

PAHs constitute a chemically diverse group of contaminants present in both environmental and food matrices, characterized by two or more fused aromatic rings that confer stability and persistence [20]. In foods, their occurrence is strongly associated with thermal treatments such as smoking, grilling, and smoke‐drying, where lignocellulosic biomass serves as the primary source of precursors. Under high temperatures, cellulose, hemicellulose, and lignin undergo pyrolytic decomposition and partial oxidation, generating a variety of radical intermediates. Instead of complete oxidation to CO_2_ and H_2_O, these reactive fragments undergo intramolecular cyclization and condensation reactions, producing stable polyaromatic ring systems [17, 21, 22]. The specific PAH profile depends on combustion conditions, fuel composition, and oxygen availability, with incomplete combustion favoring the formation of high‐molecular‐weight congeners. Because many PAHs are recognized for their mutagenic and carcinogenic properties and are subject to strict regulatory limits, systematic profiling of PAHs provides an essential complement to the assessment of chlorinated POPs when evaluating contamination pathways in smoke‐processed foods [17, 20–22].

**FIGURE 4:**
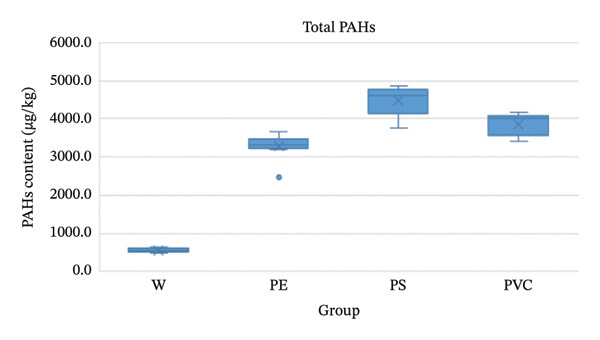
The boxplot of the total PAH contents of the samples.

In this study, the concentrations of total PAHs in smoked shiitake samples exhibited significant variation depending on the fuel type used Table S3, Figure 4. Mushrooms smoked with clean wood (W) contained the lowest levels, consistently below 650 µg/kg, representing baseline contamination derived from lignocellulosic combustion. Incorporation of plastics into the fuel markedly increased PAH burdens. The PE group showed intermediate levels, typically ranging between 2500 and 3700 µg/kg, while the PS group yielded the highest concentrations, averaging around 4500 µg/kg with several samples exceeding 4800 µg/kg. Samples smoked with PVC also displayed elevated PAHs, though with greater variability, spanning 3400–4200 µg/kg. Statistical testing confirmed that all plastic‐adulterated groups (PE, PS, and PVC) were significantly higher than W (*p* < 0.001) and that PAH contents in the PS group were significantly greater than those in PE and PVC (*p* < 0.01, Benjamini–Hochberg adjusted).

These results demonstrate that PAH accumulation follows a distinct gradient across treatments, in the order *W* < PE < PVC < PS, which parallels the trends observed for PCBs and PCDD/Fs. While wood alone contributes relatively low levels of PAHs, the presence of plastics, particularly PS, substantially enhances the generation of polycyclic aromatic contaminants, underscoring the role of fuel composition in shaping the toxicological profile of smoked foods.

**FIGURE 5:**
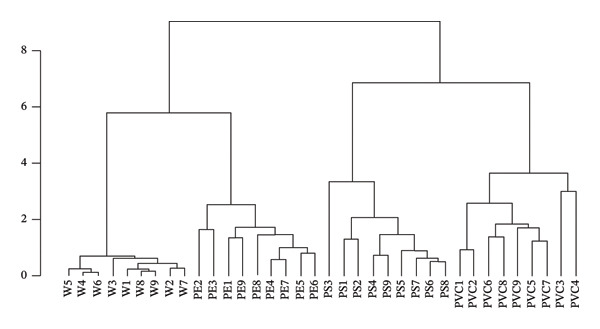
Hierarchical cluster analysis dendrogram of the PAHs in smoked shiitake samples.

HCA based on total PAH contents provided further confirmation of the trends identified in the descriptive and statistical analyses (Figure 5). The *W* samples formed a compact and well‐separated cluster, consistent with their uniformly low PAH levels and reflecting baseline contamination from wood combustion alone. In contrast, all plastic‐adulterated groups were clearly segregated from *W*, but notable differences emerged in their clustering patterns. PE and PVC samples grouped more closely together, suggesting a degree of similarity in their PAH accumulation profiles despite some variability within PVC. By comparison, the PS group formed a distinct cluster that was positioned furthest from *W*, underscoring its unique contamination pattern and the highest PAH burdens among all treatments. The tight within‐group clustering across all fuel types highlights the consistency of contaminant accumulation, while the relative distances between clusters reinforce the gradient of contamination severity: *W* < PE ≈ PVC < PS. This analysis demonstrates that although both PE and PVC enhance PAH formation compared to wood alone, the impact of PS is particularly pronounced, leading to a markedly different contamination fingerprint.

PCA of individual PAHs provided deeper insights into the compositional differences among fuel types. The scree plot and eigenvalue distribution showed that the first three principal components had eigenvalues greater than one (9.66, 3.69, and 2.85, respectively), together accounting for 95.3% of the total variance. The first two PCs explained 78.5% of the variance (PC1 = 56.8%, PC2 = 21.7%), providing sufficient resolution to distinguish the groups. The loading plot (Figure S2) indicated that PC1 was primarily driven by high‐molecular‐weight PAHs such as chrysene, benzo[a]pyrene, and indeno[1,2,3‐cd]pyrene. At the same time, PC2 was influenced by lighter compounds such as naphthalene, acenaphthene, and acenaphthylene.

**FIGURE 6:**
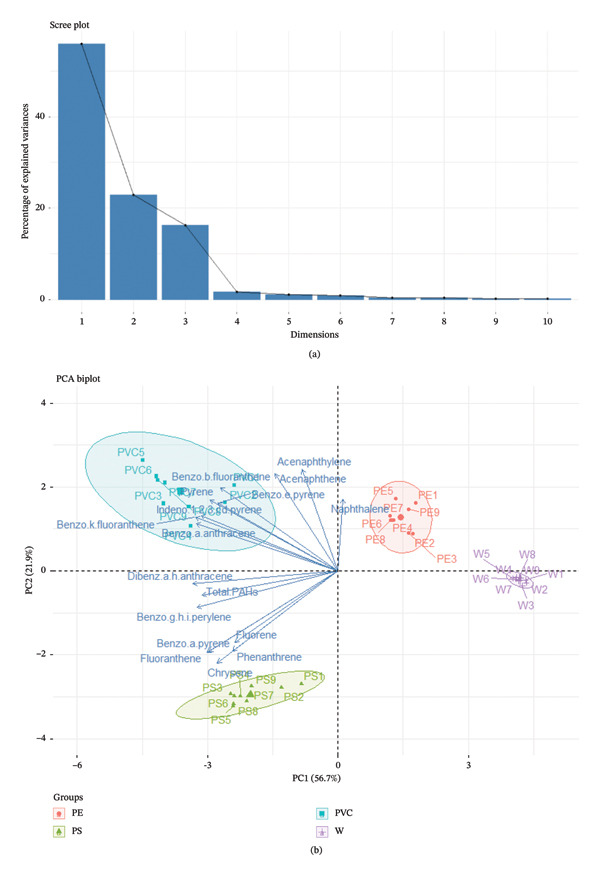
The PCA (a) scree plot and (b) biplot of PAH contents of the samples.

The PCA biplot (Figure 6) clearly separated the four groups of shiitake mushrooms smoked with different fuel materials. W samples clustered tightly with minimal variation, reflecting their uniformly low PAH levels. PE samples were positioned towards the positive axis of PC2, associated with light PAHs including naphthalene and acenaphthylene, indicating that PE combustion favored the production of lower molecular weight congeners. By contrast, PS samples were displaced along the negative axis of PC2, in proximity to fluoranthene, phenanthrene, chrysene, and benzo[a]pyrene, highlighting their enrichment in heavier carcinogenic PAHs. PVC samples clustered distinctly along the positive axis of PC1, closely aligned with benzo[b]fluoranthene, indeno[1,2,3‐cd]pyrene, and dibenz[a, h]anthracene, demonstrating that PVC combustion generated the broadest spectrum of high‐molecular‐weight PAHs.

Extending these multivariate patterns with inferential statistics, all marker compounds showed significant differences among fuel types (Kruskal–Wallis H = 28.9–32.8, *p* < 10^−6^). Light PAHs characteristic of the PE group were consistently higher in PE by median—naphthalene (PE 1397 vs PS 291 vs PVC 474 µg/kg), acenaphthylene (41.4 vs 9.8 vs 25.8), and acenaphthene (22.0 vs 10.7 vs 18.7)—with BH‐adjusted post hoc tests confirming PE > PS and PE > PVC for naphthalene and acenaphthylene (*p* ≤ 0.001), while acenaphthene showed PE > PS (*p* = 0.0007) but PE ≈ PVC (*p* = 0.112). The PS group was distinguished by higher mid‐/high‐molecular‐weight PAHs such as fluorene, phenanthrene, fluoranthene, chrysene, and benzo[a]pyrene, with all showing PS > PE and PS > PVC (*p* ≤ 0.001). For benzo[g, h, i]perylene and dibenz[a, h]anthracene, PS and PVC displayed similar medians (*p* = 0.289–0.860), indicating partial overlap. The PVC group showed the most pronounced enrichment in several high‐ring PAHs, including pyrene, benzo[a]anthracene, benzo[b]fluoranthene, benzo[k] fluoranthene, benzo[e]pyrene, benzo[a]pyrene, and indeno[1,2,3‐cd]pyrene, all with PVC > PE and PVC > PS (*p* ≤ 0.001), highlighting the distinctive contribution of PVC to complex, highly carcinogenic PAH mixtures. Importantly, all plastic‐adulterated groups differed strongly from the wood control for every compound (W vs PE, W vs PS, W vs PVC, adjusted *p* ≤ 0.001).

Together with the HCA results, PCA confirmed the grouping by fuel type and revealed characteristic PAH signatures associated with each treatment. These findings highlight that although all plastic‐adulterated fuels substantially increased PAH contamination relative to wood, the type of plastic determined the congener profile: PE favoring lighter PAHs, PS driving the accumulation of fluoranthene‐type PAHs, and PVC producing the most complex mixture dominated by high‐molecular‐weight species. This gradient of contamination severity and compositional specificity mirrors the trends observed for PCBs and PCDD/Fs, underlining the critical influence of fuel composition on the toxicological risk of smoke‐dried foods.

## 4. Discussion

The present study provides a comprehensive assessment of smoke‐derived contaminants in shiitake mushrooms subjected to traditional smoking with wood and plastic‐adulterated fuels. Comparison of the PAH dataset with PCB and PCDD/F profiles revealed both converging and diverging patterns. In all cases, the use of clean wood resulted in the lowest contaminant burdens, with PAHs restricted to background levels and PCBs/PCDD/Fs virtually absent. The introduction of plastics consistently increased contamination, but the magnitude and chemical fingerprints differed by plastic type. Similar observations have been reported in studies showing that uncontrolled combustion and waste burning are important sources of PAHs, PCBs, and PCDD/Fs in food products and the environment [23, 24].

For PAHs, PE combustion favored the generation of lighter congeners such as naphthalene, acenaphthylene, and acenaphthene, whereas PS promoted the accumulation of mid‐ to high‐molecular‐weight PAHs, including fluoranthene, chrysene, and benzo[a]pyrene. PVC combustion produced the most complex spectrum, dominated by high‐ring PAHs such as indeno[1,2,3‐cd]pyrene and benzo[b]fluoranthene. Similar effects of smoking conditions and fuel composition on PAH formation have been reported previously in smoked meat and fish products [25, 26]. A broadly similar trend was observed for chlorinated POPs: PE gave intermediate levels of PCBs with moderate contributions of dioxin‐like congeners, PS resulted in sharp increases in several coplanar PCBs and dioxins, and PVC produced the highest levels, especially of PCDD/Fs. These findings are consistent with previous reports indicating that chlorine‐containing combustion materials strongly promote the formation of DRCs [23, 24, 27]. These parallel patterns confirm that both aromatic hydrocarbons and chlorinated organics respond strongly to fuel composition, with PS and PVC posing the greatest toxicological concern. At the same time, the differences in congener specificity—light PAHs from PE, heavy PAHs from PS, and PCDD/F enrichment from PVC—underscore the distinct mechanistic pathways by which different plastics contribute to smoke contamination.

Importantly, shiitake mushrooms dried by hot air at 90°C in an oven showed no detectable PCBs or PAHs, indicating that these compounds were not intrinsic to the mushrooms or the drying process it but were introduced exclusively during smoking. This finding strengthens the conclusion that contamination arises from the combustion of fuels, particularly when plastics are present, rather than from thermal treatment alone.

In Vietnam, shiitake mushrooms are widely consumed and are commonly preserved through smoke‐drying, a traditional method valued for extending shelf life and imparting flavor. However, this is the first study to characterize how the unintentional use of plastics in smoking fuels alters contaminant profiles in smoke‐processed shiitake. The results demonstrate not only substantial increases in PAH and PCB/PCDD/F burdens when plastics are present, but also distinct congener patterns that may inform risk assessment and regulatory control. By establishing these chemical fingerprints, this work provides novel evidence that highlights the toxicological risks associated with plastic‐contaminated fuels in food processing and sets a foundation for future monitoring and policy interventions.

## 5. Conclusion

This work demonstrates that the combustion of plastics in smoking fuels markedly alters the contamination profiles of traditionally processed shiitake mushrooms. While clean wood produced only background levels of PAHs and negligible chlorinated POPs, the inclusion of PE, PS, or PVC resulted in significant and fuel‐specific increases. PE contributed primarily to lighter PAHs and moderate PCB enrichment, PS promoted the accumulation of carcinogenic high‐molecular‐weight PAHs and coplanar PCBs, and PVC generated the highest burdens overall, dominated by dioxin‐like congeners and diverse high‐ring PAHs. Statistical testing confirmed that all plastic‐adulterated fuels differed significantly from wood controls, and multivariate analyses highlighted clear clustering and pollutant fingerprints associated with each plastic type. These results emphasize the critical role of fuel composition in shaping the toxicological risk of smoke‐dried foods. Importantly, this study provides the first evidence of plastic‐induced contamination profiles in smoke‐dried shiitake mushrooms in Vietnam, where this preservation method remains widely practiced. The findings underscore the need for stronger regulation and increased awareness of fuel sources in traditional food processing to ensure food quality, protect public health, and inform future risk assessment and monitoring efforts.

## Funding

This research was supported by the Vietnam Academy of Science and Technology (VAST) under grant number NCXS 01.02/23‐25.

## Conflicts of Interest

The authors declare no conflicts of interest.

## Supporting Information

Additional supporting information can be found online in the Supporting Information section.

## Supporting information


**Supporting Information** The data that support the findings of this study are available in the supporting information section of this article. Supporting information file includes the following: (1) Reagents and chemicals for experiments. (2) Sample pretreatments and chromatographic conditions for PAHs, PCBs, and PCDD/Fs analysis. (3) Data processing analysis. (4) The PCA loading plots for PCB and PCDD/F (Figure S1) and PAH (Figure S2) contents of the samples. (5) PCB and PCDD/F (Table S1) and PAHs (Table S3) contents of the samples. (6) Benjamini–Hochberg adjusted *p* values from post hoc pairwise comparisons of total PCBs and PCDDs/Fs (Table S2) and total PAHs content (Table S4) in shiitake mushrooms smoked with different fuel types.

## Data Availability

The data that support the findings of this study are available from the corresponding author upon reasonable request.
