# Supplementary material for: Effect of Plastic Composition in the Combustion Material on the Persistent Organic Pollutant Content in Smoked Dried Shiitake Mushroom
Source: J Anal Methods Chem. 2026 Aug 4;2026:1257545. doi: 10.1155/jamc/1257545 (PMC13435889; doi:10.1155/jamc/1257545)
Supplement: Supplementary file 1 [file JAMC-2026-1257545-s001.docx]

**SUPPORTING INFORMATION**

## 1. Reagents and chemicals

### 1.1. For PAH and PCB analysis

The standard mixture of PAHs (PTM PAH Mix 2000 µg/mL with > 99.2% purity) and the isotopic labelled internal standard (IS) (EPA method 8270 IS mixture 2000µg/mL in dichloromethane) were purchased from Sigma Aldrich (Singapore) and LGC (Germany), respectively. The working standard solutions of PAHs were prepared at concentration levels 2, 5, 10, 25, 50, 100, 250, 500, 1000, and 2000 ng/mL with 200 ng/mL of all isotopically labelled IS in *n-*hexane (purity for GC-MS analysis, Merck, Singapore). Certificated reference material (CRM) standard (TCL Polynuclear Aromatic hydrocarbon mix) with 95.9% purity was collected from Supelco (Singapore).

*List of the PAHs standards*

| **No.** | **Name of compound** | **No.** | **Name of compound** |
| --- | --- | --- | --- |
| 1 | Naphthalene | 11 | Benzo[b]fluoranthene |
| 2 | Acenaphthylene | 12 | Benzo[k]fluoranthene |
| 3 | Acenaphthene | 13 | Benzo[e]pyrene |
| 4 | Fluorene | 14 | Benzo[a]pyrene |
| 5 | Phenanthrene | 15 | Indeno[1,2,3-cd]pyrene |
| 6 | Anthracene | 16 | Dibenz[a,h]anthracene |
| 7 | Fluoranthene | 17 | Benzo[g,h,i]perylene |
| 8 | Pyrene |  |  |
| 9 | Benzo[a]anthracene |  |  |
| 10 | Chrysene |  |  |

PCB standards include: a mixture of 28 congeners (WHO/NIST/NOAA) Congener list (28 PCBs) with a concentration of 10 µg/mL in *n*-hexane were purchased from Accustandard (USA, P/N: C-WNN); a mixture of PCB labeled standards (^13^C_12_ LABELED PCB MIXTURE including PCB-28, -52, -101, -138, -153, -180, -209) with a concentration of 5 µg/mL in *n*-hexane were obtained from CIL (Germany) and a mixture of 5 IS (^13^C_12_ LABELED PCB MIXTURE including PCB-77, -81, -123, -126, -169) with concentration of 1 µg/mL were supplied by LGC (Germany, P/N: EC-4938)

Solvents used for PAH and PCB analysis, such as hexane, acetone, dichloromethane, and methanol, and other chemicals such as Na_2_SO_4_, silica gel, and H_2_SO_4_ 98% being analytical grade with high purity, were collected from Merck (Singapore). Anhydrous Na_2_SO_4­_ was baked at 450^o^C for 3 hours and kept in an amber glass bottle in the desiccator.

### 1.2. For PCDD/F analysis

The set of PCDD/F standards according to the US EPA method 1613B and the US EPA method 23, manufactured by Cerilliant Corporation, Cambridge Isotope Laboratories, Inc., USA (referred to as CIL company), includes calibration solutions, isotopically fully labelled (^13^C-labelled), IS solutions (Part Numbers (P/N): EDF-9999 and EDF-4052). The IS spiking solution and labelled compound stock solution for both US EPA method 1613B and US EPA method 23 were used to determine the recovery efficiency (P/N CIL EDF-5999, EDF-8999, EDF-4053, and EDF-4055). The tuning solution, perfluorotributylamine (FC-43), was provided by Thermo Scientific (USA).

Nitrogen and helium gas with a purity of 99.99%. Solvents for PCDD/Fs analysis (acetone, dichloromethane, toluene, nonane, and n-hexane were used at dioxin analysis grade) and chemicals (anhydrous sodium sulphate, potassium hydroxide, sulphuric acid, and silver nitrate at chromatography grade) were purchased from Merck (Germany). The 60-200 mesh silica gel for open-column chromatography was collected from VWR International (USA). Activated carbon impregnated on silica gel (P/N 019-11941) was obtained by Wako (Japan).

## 2. Sample pretreatments and chromatographic conditions

The isotopic dilution gas chromatography-tandem high-resolution mass spectrometry was employed to quantify the concentrations of PAHs, PCBs, and PCDD/Fs in smoked shiitake mushroom samples. However, sample pretreatment processes and the analytical instrumentation were adjusted according to the target compounds. Detailed information for each type of analyzed POPs is provided below.

### 2.1. For PAH and PCB analysis

#### 2.1.1. Sample extraction

A 5g freeze-dried sample was taken and respectively spiked with 20 µL (10 pg/ µL) ^13^C-labelled IS of quantified PAHs and PCBs. Then, the extraction of these smoked shiitake mushroom samples was performed with an accelerated solvent extractor E-916 (Büchi), carried out with the mixture of toluene and acetone (7:3, v/v/) at 120 °C and 100 bar at a static time of 15 min. Three static cycles were accomplished.

After that, the total sample extract was divided into two parts. The first was used for fat content measurement, and the second was analyzed to determine the POPs content in the smoked shiitake mushroom sample.

#### 2.1.2. Determination of fat content

The fat content in each sample was determined by a gravimetric method. The example extract was evaporated with low pressure until the solvent was completely removed from the samples. After being dried at 80^ᵒ^C for 5 hours, the mass of the extract was measured to determine the fat content.

#### 2.1.3. Purification and enrichment

The remaining extracts were evaporated to 0.5 mL by gentle nitrogen flow, then exchanged to hexane, and cleaned up by an acidified silica gel SPE cartridge. The acidified silica cartridges were conditioned with 10 mL MeOH and then followed by 10 mL hexane at a flow rate of approximately 3 mL/min. The extracts were transferred to the SPE cartridge and eluted with 12 mL mixture of hexane/dichloromethane (1/1, v/v/). Eluent solutions were concentrated under a gentle stream of nitrogen to nearly dry and then refilled with exactly 1 mL of hexane. These samples were subjected to analysis by GC-EI-MS/MS. The concentration of PAHs in the real samples was quantified by using an isotopically labelled IS calibration curve.

#### 2.1.4. GC-MS analysis and instrumentation

A gas chromatography (GC Trace 1310, Thermo Scientific, USA) including TriPlus RSH liquid autosampler coupled with an electron impact ionization tandem mass spectrometry (Model TSQ 9000, Thermo Scientific, USA) was used for data acquisition. The sensitivity of the mass spectrometer was regularly checked by using FC-43 as a tuning solution. A Thermo DB-5MS capillary column (30 m × 0.25 mm × 0.25 µm, 5% methylphenyl polysiloxane stationary phase) was used to separate 17 PAHs and 28 PCBs. The injection volume was set to 1 µL by the liquid autosampler using the splitless mode. The temperature of the injector was kept at 280°C. Helium (99.999% purity) was used as carrier gas and constantly kept at 1.0 mL/min whole gas chromatographic separation time. These congeners were separated by the program of temperature as follows: the initial temperature was started from 100°C and kept for 3 minutes, raised linearly to 210°C at 15°C/min, then increased continuously to 300°C at a rate of 10°C/min, and finally held for 15 minutes for conditioning the GC column. The transfer line of the GC coupled with the mass spectrometer was set at 300°C. The temperature of the electron ionisation source and the quadrupole were kept constantly at 280°C and 150°C, respectively. Energy ionization was kept at 70 eV. Positive EI-MS/MS detected PAHs and PCBs in the selected reaction monitoring (SRM) mode.

### 2.2. For PCDD/F analysis

#### 2.2.1. Sample extraction

Extraction for PCDD/F analysis was followed by a protocol analogous to the extraction process designated for PAH and PCB congeners. However, before loading the sample in the E-916 accelerated solvent extractor, isotopically labeled PCDD/Fs standard solution was spiked into the freeze-dried samples at a volume of 50 µL (2 pg/µL).

The spiked ^13^C-labeled isotope PCDD/F standards were used before extraction to evaluate the extraction efficiency of the method. This procedure was carried out in accordance with Method 1613B, as stipulated by the United States Environmental Protection Agency (US EPA) in 1991.

#### 2.2.2. Purification and enrichment

The sample was placed into a multilayer column coupled with the activated carbon column to purify the sample. After that, the clean extract was continuously evaporated under lower pressure to a volume of approximately 1mL and evaporated in nitrogen gas. Then, it was mixed with 5 µL of the IS at a concentration of 2 pg/µL and made up to the volume of 10 µL with nonane.

All sample was filtered through a 0.22 µm membrane before analysis in the GC/MS system.

#### 2.2.3. GC-MS analysis and instrument

The analysis process for PCDD/Fs congeners was performed on a gas chromatographic system similar to the equipment used for the quantification of PAHs and PCBs. However, instead of electron impact tandem mass spectrometry, it was equipped with a high-resolution mass spectrometer (Magnetic Sector/Double-Focusing System, Thermo Scientific, USA). In addition, the separation of 17 PCDD/F congeners was achieved on the TG-Dioxin (60m $\times$ 0,25 mm I.D. $\times$ 0.25 µm, P/N: 122-5532) column.

They were separated by programmed temperature as follows: the temperature was kept at 140°C for 2 min, heated from 14°C to 228°C at 40°C/min and from 228°C to 290°C at 1.6°C/min, heated from 290°C to 315°C at 5°C/min, then kept at constant at 315°C for 9 min and then cooled down to the beginning of the temperature program. The solvent delay was set to 17 min. The HR/MS was regularly checked by using the tuning solution (FC-43) in terms of mass accuracy and mass resolution efficiency. The resolution of mass spectrometry was set at 10,000 at 10% of peak height. The following conditions were applied for dioxin separation and detection: selective ion monitoring mode, helium as the carrier gas at a constant flow (1.2 mL/min), splitless injection at 250°C, interface temperature at 260°C, and transfer line and ion source temperatures were 280°C and 250°C, respectively.

The SRM mode was also used to detect and quantify PCDD/Fs in this study. They were identified by relative retention times and based on two characteristic masses and the ion ratio of two ions' association with measurement uncertainty. Quantitative analysis was performed by the standard internal method, following the US EPA method 1613B and the US EPA method 23.

## 2.3. Data processing analysis

The Xcalibur software with version 4.2 (Thermo Scientific, USA) was used for data handling and data processing. Data acquisition was performed using Microsoft Excel 2016 (Microsoft Corporation, USA). Standardization and multivariate statistics (principal component analysis – PCA and hierarchical clustering analysis – HCA) were conducted on the R package (R Foundation for the Statistical Computing, Vienna, Austria).

**FIGURE**

Figure S1. The PCA loading plot of PCBs and PCDDs/Fs contents of the samples

Figure S2. The PCA loading plot of the PAH contents of the samples

# TABLES

*Table S1. PCBs and PCDDs/Fs contents of the samples*

|  | **Content (Mean ± SD, ng/kg of dried sample)** | | | | | | | |
| --- | --- | --- | --- | --- | --- | --- | --- | --- |
| **Sample** | **PCB 18** | **PCB 28** | **PCB 44** | **PCB 52** | **PCB 66** | **PCB 77** | **PCB 81** | **PCB 101** |
| **W1** | 22.20 ± 1.81 | 12.32 ± 1.90 | 10.98 ± 1.79 | 18.55 ± 3.11 | N.D. (not detected) | 11.05 ± 0.85 | N.D. | 10.80 ± 1.25 |
| **W2** | 18.04 ± 1.26 | 12.89 ± 0.96 | 8.81 ± 1.09 | 18.95 ± 2.46 | N.D. | 14.32 ± 1.29 | N.D. | 12.29 ± 1.77 |
| **W3** | 22.03 ± 3.83 | 11.30 ± 1.60 | 8.79 ± 1.50 | 16.95 ± 2.46 | N.D. | 13.00 ± 1.94 | N.D. | 10.59 ± 1.55 |
| **W4** | 20.34 ± 1.34 | 11.51 ± 1.72 | 13.80 ± 2.04 | 15.99 ± 2.37 | N.D. | 12.11 ± 1.00 | N.D. | 11.06 ± 1.12 |
| **W5** | 21.87 ± 3.49 | 10.08 ± 1.24 | 9.20 ± 1.15 | 15.61 ± 2.53 | N.D. | 11.81 ± 1.61 | N.D. | 11.33 ± 0.72 |
| **W6** | 22.80 ± 2.97 | 13.63 ± 1.11 | 13.85 ± 1.06 | 22.70 ± 3.51 | N.D. | 10.90 ± 1.72 | N.D. | 8.87 ± 1.22 |
| **W7** | 24.67 ± 2.67 | 13.15 ± 1.92 | 12.02 ± 1.94 | 17.14 ± 1.55 | N.D. | 9.65 ± 1.70 | N.D. | 10.15 ± 1.23 |
| **W8** | 18.61 ± 2.41 | 11.68 ± 1.78 | 9.18 ± 1.22 | 22.41 ± 3.13 | N.D. | 9.89 ± 1.07 | N.D. | 11.99 ± 1.76 |
| **W9** | 25.76 ± 3.34 | 11.53 ± 0.78 | 12.80 ± 1.81 | 20.27 ± 3.53 | N.D. | 9.53 ± 0.63 | N.D. | 13.13 ± 1.92 |
| **PE1** | 65.65 ± 8.01 | 164.70 ± 12.79 | 32.71 ± 5.79 | 33.03 ± 4.17 | 11.01 ± 1.29 | 11.04 ± 1.71 | 1.64 ± 0.17 | 32.93 ± 3.35 |
| **PE2** | 59.64 ± 10.30 | 174.82 ± 15.31 | 35.76 ± 6.37 | 25.93 ± 3.48 | 14.24 ± 1.70 | 10.93 ± 1.49 | 1.65 ± 0.23 | 41.81 ± 3.76 |
| **PE3** | 49.52 ± 6.05 | 172.10 ± 10.93 | 31.44 ± 4.35 | 27.43 ± 3.35 | 9.70 ± 1.53 | 12.48 ± 2.02 | 2.04 ± 0.23 | 28.00 ± 4.45 |
| **PE4** | 45.84 ± 4.62 | 128.70 ± 18.61 | 35.75 ± 3.09 | 39.34 ± 4.76 | 12.88 ± 1.77 | 7.75 ± 1.23 | 1.59 ± 0.24 | 42.00 ± 4.07 |
| **PE5** | 71.50 ± 10.84 | 140.47 ± 24.49 | 37.10 ± 2.39 | 27.46 ± 2.30 | 11.09 ± 1.91 | 14.53 ± 1.62 | 1.52 ± 0.14 | 39.58 ± 5.45 |
| **PE6** | 61.97 ± 5.35 | 210.20 ± 13.17 | 38.20 ± 3.19 | 25.77 ± 4.12 | 11.88 ± 0.92 | 9.08 ± 1.33 | 1.49 ± 0.19 | 37.05 ± 6.45 |
| **PE7** | 73.60 ± 9.45 | 160.00 ± 26.77 | 34.22 ± 5.70 | 30.56 ± 2.11 | 9.71 ± 1.27 | 9.04 ± 1.30 | 1.77 ± 0.13 | 42.09 ± 2.73 |
| **PE8** | 64.46 ± 7.80 | 130.17 ± 21.18 | 35.71 ± 3.85 | 43.06 ± 6.41 | 14.49 ± 2.33 | 13.05 ± 1.34 | 1.21 ± 0.10 | 42.12 ± 4.66 |
| **PE9** | 72.28 ± 7.02 | 124.19 ± 17.80 | 41.98 ± 4.22 | 31.16 ± 3.42 | 14.02 ± 1.34 | 12.25 ± 2.18 | 1.56 ± 0.20 | 25.87 ± 3.49 |
| **PS1** | 166.07 ± 14.54 | 120.02 ± 18.86 | 54.82 ± 5.52 | 34.08 ± 2.74 | 65.87 ± 10.75 | 22.18 ± 2.46 | 2.08 ± 0.29 | 21.92 ± 1.99 |
| **PS2** | 206.75 ± 13.61 | 158.58 ± 21.65 | 72.36 ± 11.94 | 30.57 ± 3.05 | 64.78 ± 5.24 | 26.42 ± 4.50 | 2.31 ± 0.19 | 22.22 ± 2.50 |
| **PS3** | 197.18 ± 34.55 | 125.02 ± 8.44 | 55.47 ± 4.89 | 31.76 ± 5.47 | 84.58 ± 5.95 | 25.01 ± 4.16 | 2.43 ± 0.22 | 19.55 ± 2.46 |
| **PS4** | 169.39 ± 15.77 | 93.45 ± 7.22 | 62.37 ± 5.59 | 33.84 ± 4.38 | 83.22 ± 11.80 | 25.90 ± 1.65 | 1.99 ± 0.26 | 17.08 ± 1.63 |
| **PS5** | 157.60 ± 19.62 | 158.29 ± 23.00 | 70.59 ± 8.55 | 39.00 ± 5.62 | 77.60 ± 12.23 | 21.45 ± 3.70 | 2.43 ± 0.22 | 24.46 ± 4.39 |
| **PS6** | 218.95 ± 28.42 | 141.10 ± 23.02 | 60.64 ± 8.40 | 33.76 ± 4.37 | 59.46 ± 4.07 | 18.72 ± 2.16 | 1.75 ± 0.18 | 21.49 ± 1.92 |
| **PS7** | 137.45 ± 21.12 | 101.22 ± 14.49 | 64.02 ± 11.02 | 43.40 ± 6.76 | 50.99 ± 6.24 | 21.93 ± 1.74 | 1.95 ± 0.33 | 21.60 ± 3.02 |
| **PS8** | 188.44 ± 24.08 | 108.36 ± 8.11 | 66.95 ± 8.48 | 37.93 ± 4.97 | 84.72 ± 9.49 | 28.18 ± 2.87 | 2.28 ± 0.29 | 21.03 ± 3.51 |
| **PS9** | 188.95 ± 21.45 | 131.68 ± 14.87 | 48.71 ± 3.72 | 30.09 ± 3.31 | 52.20 ± 8.11 | 26.88 ± 3.18 | 1.75 ± 0.13 | 19.97 ± 3.57 |
| **PVC1** | 110.51 ± 11.05 | 145.88 ± 18.20 | 77.29 ± 7.63 | 177.25 ± 31.08 | 55.19 ± 7.97 | 22.05 ± 1.54 | 11.03 ± 1.12 | 54.60 ± 7.19 |
| **PVC2** | 117.01 ± 8.62 | 177.38 ± 14.53 | 92.85 ± 7.75 | 124.05 ± 15.25 | 51.71 ± 5.44 | 22.46 ± 2.34 | 8.10 ± 0.57 | 46.90 ± 7.41 |
| **PVC3** | 85.68 ± 7.26 | 147.54 ± 15.85 | 84.85 ± 8.45 | 203.16 ± 28.83 | 40.37 ± 6.62 | 24.50 ± 1.98 | 13.16 ± 1.72 | 37.83 ± 2.35 |
| **PVC4** | 109.28 ± 6.84 | 114.14 ± 17.95 | 54.20 ± 4.61 | 122.96 ± 18.17 | 66.39 ± 6.00 | 22.51 ± 3.67 | 11.13 ± 1.05 | 39.61 ± 3.48 |
| **PVC5** | 127.24 ± 20.00 | 138.63 ± 18.65 | 56.14 ± 5.33 | 197.33 ± 18.01 | 63.16 ± 7.09 | 26.11 ± 4.49 | 13.51 ± 1.53 | 67.11 ± 7.91 |
| **PVC6** | 92.60 ± 12.42 | 165.87 ± 23.68 | 64.18 ± 10.35 | 199.94 ± 35.55 | 54.69 ± 6.32 | 14.95 ± 1.46 | 7.91 ± 0.80 | 66.59 ± 10.26 |
| **PVC7** | 114.56 ± 11.50 | 135.10 ± 14.99 | 55.47 ± 8.59 | 199.94 ± 25.33 | 65.84 ± 10.15 | 25.76 ± 2.98 | 8.43 ± 0.87 | 57.99 ± 8.83 |
| **PVC8** | 95.91 ± 13.57 | 131.81 ± 13.02 | 72.06 ± 11.37 | 119.79 ± 17.81 | 55.72 ± 5.18 | 24.49 ± 1.84 | 9.15 ± 0.65 | 38.17 ± 5.63 |
| **PVC9** | 95.67 ± 16.04 | 171.30 ± 27.41 | 70.96 ± 7.98 | 174.34 ± 24.38 | 37.31 ± 2.94 | 25.87 ± 3.54 | 11.90 ± 1.14 | 54.34 ± 6.92 |
|  | **Content (Mean ± SD, ng/kg of dried sample)** | | | | | | | |
| **Sample** | **PCB 118** | **PCB 114** | **PCB 123** | **PCB 126** | **PCB 138** | **PCB 153** | **PCB 180** | **PCB 209** |
| **W1** | N.D. | 11.02 ± 1.07 | N.D. | N.D. | 11.04 ± 1.51 | N.D. | 10.99 ± 1.63 | 11.06 ± 0.96 |
| **W2** | N.D. | 12.10 ± 1.49 | N.D. | N.D. | 12.00 ± 1.77 | N.D. | 12.80 ± 1.61 | 11.54 ± 1.96 |
| **W3** | N.D. | 10.21 ± 1.44 | N.D. | N.D. | 12.51 ± 2.10 | N.D. | 12.00 ± 0.91 | 14.62 ± 1.29 |
| **W4** | N.D. | 13.18 ± 1.53 | N.D. | N.D. | 13.37 ± 1.02 | N.D. | 9.71 ± 0.72 | 11.36 ± 0.83 |
| **W5** | N.D. | 13.88 ± 0.95 | N.D. | N.D. | 13.44 ± 1.11 | N.D. | 14.33 ± 1.95 | 12.67 ± 1.84 |
| **W6** | N.D. | 14.40 ± 0.91 | N.D. | N.D. | 8.82 ± 0.69 | N.D. | 9.13 ± 1.61 | 8.53 ± 1.36 |
| **W7** | N.D. | 11.31 ± 1.04 | N.D. | N.D. | 9.96 ± 1.40 | N.D. | 10.56 ± 1.04 | 11.58 ± 1.19 |
| **W8** | N.D. | 13.59 ± 1.18 | N.D. | N.D. | 10.96 ± 0.69 | N.D. | 11.74 ± 1.26 | 10.76 ± 1.85 |
| **W9** | N.D. | 12.86 ± 0.80 | N.D. | N.D. | 9.94 ± 1.06 | N.D. | 13.42 ± 2.01 | 14.09 ± 2.42 |
| **PE1** | 43.94 ± 5.10 | 10.94 ± 0.79 | N.D. | 4.39 ± 0.65 | 21.90 ± 2.99 | 10.96 ± 1.76 | 44.26 ± 7.68 | 11.05 ± 1.55 |
| **PE2** | 34.35 ± 5.81 | 7.34 ± 1.24 | N.D. | 5.27 ± 0.74 | 23.87 ± 1.95 | 12.67 ± 1.65 | 42.23 ± 3.41 | 14.36 ± 1.90 |
| **PE3** | 37.83 ± 3.09 | 9.89 ± 1.02 | N.D. | 5.41 ± 0.37 | 23.30 ± 2.58 | 13.23 ± 0.88 | 51.57 ± 3.46 | 8.29 ± 1.01 |
| **PE4** | 37.84 ± 2.47 | 12.58 ± 1.74 | N.D. | 3.70 ± 0.53 | 24.65 ± 3.13 | 9.88 ± 1.54 | 52.72 ± 7.25 | 7.40 ± 0.94 |
| **PE5** | 57.41 ± 9.85 | 10.11 ± 1.45 | N.D. | 4.15 ± 0.39 | 15.03 ± 2.39 | 11.60 ± 1.61 | 50.05 ± 8.80 | 13.33 ± 2.23 |
| **PE6** | 36.30 ± 2.89 | 8.58 ± 1.48 | N.D. | 5.51 ± 0.78 | 21.16 ± 3.32 | 12.79 ± 1.90 | 36.05 ± 4.29 | 10.28 ± 0.97 |
| **PE7** | 41.21 ± 4.56 | 11.94 ± 1.09 | N.D. | 3.31 ± 0.24 | 15.27 ± 1.48 | 11.26 ± 0.70 | 31.89 ± 2.36 | 11.14 ± 1.98 |
| **PE8** | 38.57 ± 3.83 | 8.17 ± 0.54 | N.D. | 5.45 ± 0.86 | 22.50 ± 3.12 | 11.57 ± 0.82 | 30.37 ± 3.57 | 13.89 ± 1.91 |
| **PE9** | 34.50 ± 5.25 | 8.02 ± 0.73 | N.D. | 5.40 ± 0.94 | 20.91 ± 3.47 | 7.61 ± 0.84 | 51.42 ± 8.05 | 11.59 ± 1.97 |
| **PS1** | 22.18 ± 3.37 | 10.96 ± 0.78 | N.D. | 13.10 ± 2.17 | 22.14 ± 2.41 | 11.03 ± 1.92 | 55.03 ± 4.59 | 33.20 ± 4.75 |
| **PS2** | 25.47 ± 3.44 | 13.10 ± 1.10 | N.D. | 17.44 ± 2.98 | 27.54 ± 3.69 | 14.25 ± 2.54 | 57.19 ± 6.93 | 26.61 ± 3.56 |
| **PS3** | 26.54 ± 3.04 | 10.57 ± 1.20 | N.D. | 10.47 ± 0.96 | 26.52 ± 2.27 | 10.57 ± 0.86 | 49.13 ± 3.20 | 43.67 ± 3.42 |
| **PS4** | 21.88 ± 1.98 | 9.34 ± 0.89 | N.D. | 15.41 ± 1.87 | 22.18 ± 3.77 | 12.22 ± 0.85 | 47.99 ± 6.34 | 38.32 ± 3.87 |
| **PS5** | 27.70 ± 3.51 | 13.26 ± 1.74 | N.D. | 14.93 ± 1.32 | 26.99 ± 4.38 | 9.42 ± 0.97 | 64.82 ± 6.49 | 33.57 ± 3.81 |
| **PS6** | 24.29 ± 3.25 | 10.29 ± 0.70 | N.D. | 17.29 ± 2.62 | 25.15 ± 3.12 | 8.58 ± 1.46 | 52.41 ± 9.09 | 37.32 ± 2.41 |
| **PS7** | 22.31 ± 2.31 | 13.51 ± 2.24 | N.D. | 11.06 ± 1.47 | 20.12 ± 1.80 | 11.99 ± 1.58 | 57.39 ± 5.44 | 32.83 ± 4.41 |
| **PS8** | 28.74 ± 4.78 | 14.49 ± 2.14 | N.D. | 13.74 ± 0.86 | 21.17 ± 1.56 | 8.56 ± 0.93 | 56.38 ± 3.53 | 38.00 ± 3.81 |
| **PS9** | 24.45 ± 4.28 | 13.14 ± 1.74 | N.D. | 15.79 ± 1.36 | 27.32 ± 3.66 | 13.48 ± 1.13 | 55.20 ± 9.84 | 39.27 ± 3.93 |
| **PVC1** | 88.18 ± 11.28 | 55.28 ± 6.98 | 44.29 ± 5.70 | 9.91 ± 1.20 | 32.71 ± 3.36 | 21.81 ± 2.53 | 121.88 ± 13.27 | 36.52 ± 6.27 |
| **PVC2** | 81.76 ± 9.37 | 57.95 ± 9.90 | 38.59 ± 3.30 | 9.49 ± 0.68 | 23.82 ± 1.95 | 23.42 ± 4.02 | 127.27 ± 8.03 | 33.95 ± 2.25 |
| **PVC3** | 61.44 ± 4.57 | 58.48 ± 5.99 | 49.82 ± 7.40 | 8.83 ± 1.45 | 26.55 ± 2.70 | 22.21 ± 2.61 | 125.39 ± 17.62 | 42.70 ± 4.16 |
| **PVC4** | 70.66 ± 8.61 | 43.38 ± 3.94 | 35.94 ± 5.70 | 11.14 ± 1.10 | 29.37 ± 4.48 | 24.27 ± 4.17 | 132.85 ± 16.84 | 34.11 ± 5.10 |
| **PVC5** | 101.60 ± 16.24 | 39.17 ± 4.08 | 50.17 ± 8.99 | 7.61 ± 0.53 | 23.69 ± 2.48 | 24.70 ± 2.44 | 104.75 ± 7.79 | 30.46 ± 2.81 |
| **PVC6** | 86.81 ± 13.63 | 64.83 ± 7.90 | 47.28 ± 8.16 | 8.54 ± 0.74 | 35.15 ± 4.26 | 18.30 ± 1.65 | 125.84 ± 21.83 | 25.85 ± 4.45 |
| **PVC7** | 103.38 ± 16.58 | 48.41 ± 3.56 | 43.41 ± 6.42 | 11.54 ± 1.57 | 39.97 ± 3.14 | 17.46 ± 2.15 | 143.67 ± 22.97 | 44.37 ± 3.07 |
| **PVC8** | 99.72 ± 8.39 | 40.03 ± 2.64 | 50.44 ± 3.50 | 8.23 ± 0.69 | 36.38 ± 5.26 | 23.82 ± 2.74 | 127.71 ± 9.35 | 30.69 ± 3.33 |
| **PVC9** | 77.93 ± 9.91 | 58.78 ± 8.14 | 34.71 ± 5.50 | 8.23 ± 0.55 | 22.73 ± 1.98 | 18.98 ± 2.35 | 99.98 ± 11.90 | 34.43 ± 3.07 |
|  | **Content (Mean ± SD, ng/kg of dried sample)** | | | | | | | |
| **Sample** | **123478-HxCDD** | **123678-HxCDD** | **1234678-HpCDD** | **OCDD** | **2378-TCDF** | **12378-PeCDF** | **123478-HxCDF** | **123678-HxCDF** |
| **W1** | N.D. | N.D. | N.D. | N.D. | N.D. | N.D. | N.D. | N.D. |
| **W2** | N.D. | N.D. | N.D. | N.D. | N.D. | N.D. | N.D. | N.D. |
| **W3** | N.D. | N.D. | N.D. | N.D. | N.D. | N.D. | N.D. | N.D. |
| **W4** | N.D. | N.D. | N.D. | N.D. | N.D. | N.D. | N.D. | N.D. |
| **W5** | N.D. | N.D. | N.D. | N.D. | N.D. | N.D. | N.D. | N.D. |
| **W6** | N.D. | N.D. | N.D. | N.D. | N.D. | N.D. | N.D. | N.D. |
| **W7** | N.D. | N.D. | N.D. | N.D. | N.D. | N.D. | N.D. | N.D. |
| **W8** | N.D. | N.D. | N.D. | N.D. | N.D. | N.D. | N.D. | N.D. |
| **W9** | N.D. | N.D. | N.D. | N.D. | N.D. | N.D. | N.D. | N.D. |
| **PE1** | N.D. | N.D. | N.D. | N.D. | N.D. | 0.35 ± 0.05 | N.D. | 0.45 ± 0.05 |
| **PE2** | N.D. | N.D. | N.D. | N.D. | N.D. | 0.40 ± 0.03 | N.D. | 0.44 ± 0.07 |
| **PE3** | N.D. | N.D. | N.D. | N.D. | N.D. | 0.34 ± 0.04 | N.D. | 0.59 ± 0.08 |
| **PE4** | N.D. | N.D. | N.D. | N.D. | N.D. | 0.40 ± 0.06 | N.D. | 0.34 ± 0.04 |
| **PE5** | N.D. | N.D. | N.D. | N.D. | N.D. | 0.26 ± 0.03 | N.D. | 0.37 ± 0.04 |
| **PE6** | N.D. | N.D. | N.D. | N.D. | N.D. | 0.27 ± 0.03 | N.D. | 0.31 ± 0.03 |
| **PE7** | N.D. | N.D. | N.D. | N.D. | N.D. | 0.35 ± 0.03 | N.D. | 0.59 ± 0.04 |
| **PE8** | N.D. | N.D. | N.D. | N.D. | N.D. | 0.30 ± 0.05 | N.D. | 0.43 ± 0.08 |
| **PE9** | N.D. | N.D. | N.D. | N.D. | N.D. | 0.43 ± 0.07 | N.D. | 0.35 ± 0.04 |
| **PS1** | N.D. | 0.40 ± 0.03 | 0.25 ± 0.03 | 0.70 ± 0.09 | 0.80 ± 0.14 | 0.55 ± 0.09 | N.D. | N.D. |
| **PS2** | N.D. | 0.45 ± 0.08 | 0.30 ± 0.03 | 0.77 ± 0.12 | 0.91 ± 0.09 | 0.68 ± 0.08 | N.D. | N.D. |
| **PS3** | N.D. | 0.49 ± 0.09 | 0.30 ± 0.03 | 0.66 ± 0.06 | 0.91 ± 0.15 | 0.51 ± 0.09 | N.D. | N.D. |
| **PS4** | N.D. | 0.37 ± 0.05 | 0.23 ± 0.03 | 0.80 ± 0.08 | 0.65 ± 0.12 | 0.49 ± 0.08 | N.D. | N.D. |
| **PS5** | N.D. | 0.31 ± 0.05 | 0.26 ± 0.02 | 0.86 ± 0.08 | 0.98 ± 0.10 | 0.50 ± 0.04 | N.D. | N.D. |
| **PS6** | N.D. | 0.36 ± 0.03 | 0.31 ± 0.04 | 0.81 ± 0.08 | 0.74 ± 0.05 | 0.56 ± 0.05 | N.D. | N.D. |
| **PS7** | N.D. | 0.51 ± 0.06 | 0.30 ± 0.03 | 0.76 ± 0.13 | 0.72 ± 0.07 | 0.62 ± 0.06 | N.D. | N.D. |
| **PS8** | N.D. | 0.44 ± 0.04 | 0.29 ± 0.04 | 0.90 ± 0.12 | 0.95 ± 0.16 | 0.54 ± 0.05 | N.D. | N.D. |
| **PS9** | N.D. | 0.36 ± 0.04 | 0.28 ± 0.02 | 0.86 ± 0.10 | 0.81 ± 0.13 | 0.62 ± 0.06 | N.D. | N.D. |
| **PVC1** | 1.20 ± 0.14 | 0.35 ± 0.04 | 0.55 ± 0.07 | 0.94 ± 0.15 | 1.34 ± 0.16 | 1.55 ± 0.17 | 0.50 ± 0.06 | 0.40 ± 0.07 |
| **PVC2** | 0.94 ± 0.13 | 0.28 ± 0.02 | 0.57 ± 0.08 | 0.97 ± 0.08 | 1.36 ± 0.11 | 1.79 ± 0.16 | 0.38 ± 0.03 | 0.35 ± 0.03 |
| **PVC3** | 0.89 ± 0.15 | 0.30 ± 0.03 | 0.56 ± 0.05 | 0.77 ± 0.08 | 0.91 ± 0.13 | 1.06 ± 0.13 | 0.47 ± 0.06 | 0.31 ± 0.04 |
| **PVC4** | 0.91 ± 0.07 | 0.38 ± 0.05 | 0.45 ± 0.05 | 0.85 ± 0.14 | 1.56 ± 0.14 | 1.25 ± 0.22 | 0.54 ± 0.07 | 0.44 ± 0.07 |
| **PVC5** | 1.40 ± 0.23 | 0.36 ± 0.05 | 0.44 ± 0.05 | 0.92 ± 0.14 | 1.52 ± 0.21 | 1.75 ± 0.18 | 0.50 ± 0.06 | 0.32 ± 0.03 |
| **PVC6** | 1.38 ± 0.10 | 0.36 ± 0.05 | 0.47 ± 0.05 | 0.80 ± 0.05 | 1.44 ± 0.11 | 1.65 ± 0.20 | 0.53 ± 0.06 | 0.34 ± 0.05 |
| **PVC7** | 1.11 ± 0.14 | 0.28 ± 0.04 | 0.45 ± 0.06 | 0.88 ± 0.11 | 1.18 ± 0.20 | 1.41 ± 0.17 | 0.34 ± 0.06 | 0.48 ± 0.03 |
| **PVC8** | 0.88 ± 0.07 | 0.38 ± 0.03 | 0.67 ± 0.10 | 0.83 ± 0.09 | 0.99 ± 0.08 | 1.85 ± 0.20 | 0.42 ± 0.04 | 0.32 ± 0.02 |
| **PVC9** | 1.46 ± 0.23 | 0.37 ± 0.06 | 0.41 ± 0.04 | 0.69 ± 0.08 | 1.53 ± 0.27 | 1.17 ± 0.09 | 0.59 ± 0.10 | 0.42 ± 0.03 |
|  | **Content (Mean ± SD, ng/kg of dried sample)** | | | | |  |  |  |
| **Sample** | **123789-HxCDF** | **234678-HxCDF** | **1234678-HpCDF** | **Total PCBs** | **Total PCDD/Fs** |  |  |  |
| **W1** | N.D. | N.D. | N.D. | 130.01 ± 13.55 | N.D. |  |  |  |
| **W2** | N.D. | N.D. | N.D. | 133.74 ± 17.50 | N.D. |  |  |  |
| **W3** | N.D. | N.D. | N.D. | 132.00 ± 17.20 | N.D. |  |  |  |
| **W4** | N.D. | N.D. | N.D. | 132.43 ± 15.59 | N.D. |  |  |  |
| **W5** | N.D. | N.D. | N.D. | 134.22 ± 16.34 | N.D. |  |  |  |
| **W6** | N.D. | N.D. | N.D. | 133.64 ± 17.38 | N.D. |  |  |  |
| **W7** | N.D. | N.D. | N.D. | 130.20 ± 15.85 | N.D. |  |  |  |
| **W8** | N.D. | N.D. | N.D. | 130.81 ± 15.78 | N.D. |  |  |  |
| **W9** | N.D. | N.D. | N.D. | 143.34 ± 17.28 | N.D. |  |  |  |
| **PE1** | N.D. | N.D. | N.D. | 500.15 ± 54.66 | 0.80 ± 0.07 |  |  |  |
| **PE2** | N.D. | N.D. | N.D. | 504.87 ± 53.37 | 0.84 ± 0.11 |  |  |  |
| **PE3** | N.D. | N.D. | N.D. | 482.22 ± 61.91 | 0.93 ± 0.07 |  |  |  |
| **PE4** | N.D. | N.D. | N.D. | 462.60 ± 53.24 | 0.74 ± 0.10 |  |  |  |
| **PE5** | N.D. | N.D. | N.D. | 504.93 ± 64.71 | 0.63 ± 0.11 |  |  |  |
| **PE6** | N.D. | N.D. | N.D. | 526.31 ± 72.46 | 0.58 ± 0.06 |  |  |  |
| **PE7** | N.D. | N.D. | N.D. | 487.03 ± 47.89 | 0.93 ± 0.11 |  |  |  |
| **PE8** | N.D. | N.D. | N.D. | 474.78 ± 57.96 | 0.73 ± 0.08 |  |  |  |
| **PE9** | N.D. | N.D. | N.D. | 462.75 ± 55.58 | 0.78 ± 0.08 |  |  |  |
| **PS1** | 0.20 ± 0.03 | N.D. | N.D. | 654.68 ± 57.62 | 2.89 ± 0.35 |  |  |  |
| **PS2** | 0.16 ± 0.02 | N.D. | N.D. | 765.61 ± 93.43 | 3.27 ± 0.31 |  |  |  |
| **PS3** | 0.25 ± 0.02 | N.D. | N.D. | 718.46 ± 100.05 | 3.12 ± 0.49 |  |  |  |
| **PS4** | 0.17 ± 0.03 | N.D. | N.D. | 654.58 ± 87.43 | 2.70 ± 0.36 |  |  |  |
| **PS5** | 0.19 ± 0.02 | N.D. | N.D. | 742.11 ± 98.28 | 3.10 ± 0.34 |  |  |  |
| **PS6** | 0.17 ± 0.02 | N.D. | N.D. | 731.20 ± 82.75 | 2.94 ± 0.37 |  |  |  |
| **PS7** | 0.18 ± 0.01 | N.D. | N.D. | 611.78 ± 83.47 | 3.09 ± 0.40 |  |  |  |
| **PS8** | 0.17 ± 0.02 | N.D. | N.D. | 718.96 ± 77.29 | 3.28 ± 0.41 |  |  |  |
| **PS9** | 0.25 ± 0.02 | N.D. | N.D. | 688.88 ± 94.69 | 3.19 ± 0.48 |  |  |  |
| **PVC1** | 1.15 ± 0.12 | 0.50 ± 0.04 | 0.99 ± 0.15 | 1064.37 ± 147.51 | 9.47 ± 1.08 |  |  |  |
| **PVC2** | 1.29 ± 0.13 | 0.55 ± 0.08 | 1.12 ± 0.14 | 1036.70 ± 115.75 | 9.61 ± 1.18 |  |  |  |
| **PVC3** | 1.29 ± 0.20 | 0.52 ± 0.04 | 1.10 ± 0.07 | 1032.53 ± 140.38 | 8.17 ± 1.18 |  |  |  |
| **PVC4** | 1.21 ± 0.16 | 0.42 ± 0.04 | 0.69 ± 0.05 | 921.94 ± 103.76 | 8.71 ± 0.97 |  |  |  |
| **PVC5** | 1.15 ± 0.11 | 0.46 ± 0.04 | 1.17 ± 0.08 | 1071.40 ± 133.31 | 9.98 ± 1.16 |  |  |  |
| **PVC6** | 1.40 ± 0.11 | 0.41 ± 0.04 | 1.06 ± 0.10 | 1079.34 ± 122.56 | 9.84 ± 1.22 |  |  |  |
| **PVC7** | 0.90 ± 0.15 | 0.59 ± 0.08 | 1.17 ± 0.20 | 1115.32 ± 152.49 | 8.80 ± 0.86 |  |  |  |
| **PVC8** | 1.05 ± 0.11 | 0.56 ± 0.08 | 0.80 ± 0.08 | 964.10 ± 115.03 | 8.74 ± 1.11 |  |  |  |
| **PVC9** | 1.16 ± 0.16 | 0.45 ± 0.06 | 0.88 ± 0.14 | 997.48 ± 114.73 | 9.13 ± 1.19 |  |  |  |

Table S2. Benjamini–Hochberg adjusted p-values from post-hoc pairwise comparisons of total PCBs and PCDDs/Fs in shiitake mushrooms smoked with different fuel types. All comparisons between groups were statistically significant (adjusted p < 0.001), confirming a clear stepwise gradient of contamination levels in the order W < PE < PS < PVC.

|  | Adjusted p-value (PCBs) | Adjusted p-value (PCDDs/Fs) |
| --- | --- | --- |
| W vs PE | 0.000412295 | 0.00040946 |
| W vs PS | 0.000412295 | 0.00040946 |
| W vs PVC | 0.000412295 | 0.00040946 |
| PE vs PS | 0.000412295 | 0.00040946 |
| PE vs PVC | 0.000412295 | 0.00040946 |
| PS vs PVC | 0.000412295 | 0.00040946 |

*Table S3. PAHs contents of the samples*

| **Sample** | **Content (Mean ± SD, µg/kg of dried sample)** | | | | | |
| --- | --- | --- | --- | --- | --- | --- |
|  | **Naphthalene** | **Acenaphthylene** | **Acenaphthene** | **Fluorene** | **Phenanthrene** | **Fluoranthene** |
| **W1** | 114.75 ± 9.10 | 3.48 ± 0.46 | 7.47 ± 1.29 | 20.24 ± 1.26 | 314.15 ± 26.33 | 28.13 ± 2.77 |
| **W2** | 106.19 ± 16.85 | 3.34 ± 0.21 | 5.66 ± 0.52 | 17.62 ± 2.22 | 272.88 ± 34.49 | 19.16 ± 2.60 |
| **W3** | 100.97 ± 9.19 | 1.11 ± 0.20 | 4.62 ± 0.81 | 13.06 ± 1.90 | 273.47 ± 32.31 | 17.02 ± 2.78 |
| **W4** | 110.21 ± 15.38 | 4.00 ± 0.39 | 8.14 ± 0.73 | 19.35 ± 2.40 | 270.06 ± 41.82 | 27.39 ± 1.70 |
| **W5** | 136.75 ± 24.11 | 3.48 ± 0.43 | 7.89 ± 1.12 | 19.05 ± 2.19 | 369.13 ± 61.02 | 23.43 ± 3.67 |
| **W6** | 106.57 ± 7.16 | 3.16 ± 0.41 | 8.03 ± 1.33 | 24.15 ± 2.69 | 281.58 ± 50.23 | 26.41 ± 3.31 |
| **W7** | 134.02 ± 14.94 | 4.06 ± 0.59 | 6.67 ± 0.87 | 25.29 ± 3.64 | 355.05 ± 33.23 | 27.49 ± 4.86 |
| **W8** | 135.28 ± 22.23 | 4.31 ± 0.48 | 8.13 ± 0.79 | 17.16 ± 2.75 | 341.62 ± 27.49 | 27.22 ± 2.99 |
| **W9** | 115.83 ± 17.39 | 4.31 ± 0.55 | 7.31 ± 0.88 | 17.50 ± 1.41 | 277.55 ± 20.10 | 28.60 ± 3.39 |
| **PE1** | 1517.14 ± 200.97 | 37.65 ± 2.83 | 27.38 ± 1.99 | 126.96 ± 10.65 | 1461.35 ± 115.48 | 76.27 ± 8.51 |
| **PE2** | 1402.94 ± 251.36 | 36.70 ± 6.52 | 12.67 ± 1.42 | 122.27 ± 10.42 | 1238.50 ± 191.01 | 106.92 ± 11.54 |
| **PE3** | 834.62 ± 129.32 | 29.80 ± 4.34 | 18.16 ± 2.90 | 113.75 ± 8.37 | 1038.76 ± 152.68 | 112.96 ± 11.76 |
| **PE4** | 1396.96 ± 195.73 | 47.67 ± 7.92 | 19.22 ± 1.42 | 159.12 ± 12.61 | 1637.37 ± 206.61 | 113.62 ± 17.04 |
| **PE5** | 1437.58 ± 194.47 | 45.56 ± 6.02 | 24.34 ± 3.82 | 151.50 ± 22.90 | 1288.55 ± 196.97 | 95.20 ± 16.79 |
| **PE6** | 1154.39 ± 205.36 | 44.86 ± 8.07 | 23.44 ± 1.53 | 151.54 ± 20.99 | 1536.20 ± 109.31 | 109.47 ± 14.11 |
| **PE7** | 1418.32 ± 96.75 | 41.36 ± 3.74 | 20.17 ± 3.03 | 156.68 ± 15.33 | 1481.45 ± 253.54 | 113.23 ± 11.01 |
| **PE8** | 1217.47 ± 136.02 | 33.72 ± 5.53 | 25.20 ± 2.03 | 152.96 ± 11.95 | 1400.01 ± 234.90 | 113.08 ± 19.21 |
| **PE9** | 1281.68 ± 222.26 | 43.03 ± 3.47 | 21.95 ± 2.84 | 147.32 ± 22.28 | 1405.85 ± 164.99 | 96.47 ± 11.34 |
| **PS1** | 303.93 ± 38.22 | 9.83 ± 1.03 | 10.01 ± 1.43 | 201.94 ± 29.53 | 2556.52 ± 190.52 | 592.81 ± 91.99 |
| **PS2** | 291.49 ± 35.50 | 7.28 ± 0.61 | 9.70 ± 1.56 | 222.80 ± 30.38 | 2254.25 ± 398.27 | 670.79 ± 110.16 |
| **PS3** | 224.46 ± 35.42 | 6.26 ± 0.91 | 8.77 ± 0.99 | 209.24 ± 13.09 | 1727.28 ± 231.85 | 752.32 ± 77.26 |
| **PS4** | 252.38 ± 40.67 | 10.83 ± 0.92 | 10.48 ± 0.80 | 255.40 ± 32.05 | 2684.55 ± 275.82 | 691.70 ± 83.69 |
| **PS5** | 269.99 ± 48.60 | 7.98 ± 1.43 | 12.81 ± 2.19 | 270.91 ± 32.38 | 2669.48 ± 403.82 | 695.80 ± 93.00 |
| **PS6** | 323.51 ± 44.87 | 11.22 ± 0.78 | 12.34 ± 1.85 | 220.08 ± 38.17 | 2766.21 ± 370.48 | 784.80 ± 77.25 |
| **PS7** | 244.82 ± 33.28 | 9.84 ± 1.65 | 10.79 ± 1.44 | 231.88 ± 37.47 | 2493.53 ± 255.89 | 788.90 ± 82.17 |
| **PS8** | 326.41 ± 21.16 | 9.94 ± 0.68 | 11.93 ± 1.15 | 226.86 ± 39.53 | 2587.58 ± 276.74 | 850.50 ± 129.18 |
| **PS9** | 331.62 ± 22.04 | 7.82 ± 1.40 | 10.72 ± 1.59 | 246.25 ± 33.33 | 2730.24 ± 290.86 | 614.30 ± 39.75 |
| **PVC1** | 419.97 ± 64.81 | 29.24 ± 3.47 | 16.42 ± 1.18 | 121.97 ± 21.61 | 1208.07 ± 90.33 | 375.23 ± 41.11 |
| **PVC2** | 416.10 ± 67.52 | 22.88 ± 3.91 | 17.01 ± 1.53 | 111.51 ± 17.63 | 1128.59 ± 78.35 | 461.30 ± 43.06 |
| **PVC3** | 322.58 ± 55.07 | 13.67 ± 1.37 | 22.96 ± 1.85 | 66.23 ± 11.18 | 1277.37 ± 142.45 | 458.60 ± 63.16 |
| **PVC4** | 504.30 ± 47.82 | 25.68 ± 4.37 | 16.18 ± 1.25 | 128.82 ± 9.67 | 1558.09 ± 186.63 | 506.13 ± 63.05 |
| **PVC5** | 513.35 ± 36.00 | 33.33 ± 5.28 | 19.70 ± 2.86 | 132.06 ± 21.04 | 1366.79 ± 123.20 | 411.53 ± 58.60 |
| **PVC6** | 394.30 ± 66.14 | 31.81 ± 2.63 | 18.65 ± 2.92 | 115.34 ± 15.31 | 1150.23 ± 156.99 | 473.39 ± 49.67 |
| **PVC7** | 475.66 ± 61.15 | 25.79 ± 2.80 | 19.35 ± 3.17 | 143.29 ± 24.00 | 1374.31 ± 120.33 | 440.75 ± 58.67 |
| **PVC8** | 473.94 ± 78.88 | 28.15 ± 4.60 | 20.94 ± 1.93 | 146.39 ± 25.67 | 1282.02 ± 223.17 | 556.44 ± 63.11 |
| **PVC9** | 497.02 ± 55.93 | 25.31 ± 3.40 | 18.27 ± 1.62 | 114.22 ± 18.64 | 1580.80 ± 195.18 | 426.26 ± 36.58 |
| **Sample** | **Content (Mean ± SD, µg/kg of dried sample)** | | | | | |
|  | **Pyrene** | **Benzo[a]anthracene** | **Chrysene** | **Benzo[b]fluoranthene** | **Benzo[k]fluoranthene** | **Benzo[e]pyrene** |
| **W1** | 8.98 ± 1.57 | 7.77 ± 0.96 | 9.02 ± 0.67 | 11.01 ± 0.83 | 6.40 ± 0.80 | 1.60 ± 0.17 |
| **W2** | 16.08 ± 1.27 | 9.32 ± 1.12 | 12.90 ± 1.86 | 10.90 ± 1.05 | 8.30 ± 1.35 | 1.30 ± 0.19 |
| **W3** | 21.98 ± 1.78 | 17.34 ± 1.86 | 15.87 ± 2.66 | 11.03 ± 0.91 | 9.58 ± 0.77 | 2.98 ± 0.24 |
| **W4** | 15.74 ± 2.50 | 15.33 ± 1.39 | 12.93 ± 1.51 | 14.13 ± 2.19 | 7.26 ± 0.86 | 2.09 ± 0.22 |
| **W5** | 19.35 ± 3.37 | 11.50 ± 1.61 | 11.83 ± 1.26 | 13.26 ± 1.60 | 10.31 ± 1.14 | 2.47 ± 0.22 |
| **W6** | 15.71 ± 2.75 | 14.42 ± 1.77 | 11.67 ± 1.16 | 12.52 ± 0.87 | 8.43 ± 0.71 | 1.82 ± 0.27 |
| **W7** | 20.94 ± 2.69 | 13.76 ± 2.44 | 11.60 ± 1.07 | 10.16 ± 1.24 | 10.21 ± 0.88 | 1.87 ± 0.30 |
| **W8** | 20.50 ± 3.21 | 13.44 ± 0.94 | 15.75 ± 2.43 | 10.08 ± 1.34 | 8.81 ± 0.94 | 2.24 ± 0.19 |
| **W9** | 19.65 ± 3.23 | 13.48 ± 0.84 | 14.60 ± 2.28 | 14.49 ± 2.43 | 7.86 ± 0.52 | 2.04 ± 0.32 |
| **PE1** | 41.69 ± 7.29 | 32.65 ± 5.75 | 45.27 ± 7.65 | 18.88 ± 1.81 | 24.92 ± 3.26 | 11.15 ± 0.94 |
| **PE2** | 58.43 ± 6.30 | 43.48 ± 5.36 | 57.80 ± 8.30 | 32.20 ± 2.64 | 35.75 ± 4.37 | 7.01 ± 0.96 |
| **PE3** | 80.92 ± 8.25 | 48.87 ± 6.41 | 62.47 ± 9.04 | 56.99 ± 7.99 | 36.77 ± 5.33 | 5.60 ± 0.96 |
| **PE4** | 77.83 ± 5.40 | 51.95 ± 7.13 | 56.40 ± 8.85 | 44.53 ± 6.56 | 30.04 ± 2.42 | 9.44 ± 0.94 |
| **PE5** | 75.12 ± 9.31 | 47.45 ± 3.97 | 54.50 ± 4.05 | 46.94 ± 6.01 | 36.12 ± 6.32 | 8.00 ± 0.78 |
| **PE6** | 68.32 ± 9.32 | 38.78 ± 5.89 | 63.51 ± 5.25 | 33.63 ± 2.40 | 35.94 ± 3.36 | 7.98 ± 0.67 |
| **PE7** | 70.29 ± 6.01 | 40.53 ± 7.06 | 65.35 ± 8.20 | 44.90 ± 7.90 | 33.87 ± 3.84 | 10.33 ± 0.72 |
| **PE8** | 75.96 ± 7.32 | 46.18 ± 8.26 | 67.09 ± 10.98 | 40.88 ± 4.91 | 31.95 ± 3.19 | 9.15 ± 1.42 |
| **PE9** | 76.02 ± 7.53 | 38.71 ± 3.37 | 56.93 ± 9.20 | 47.60 ± 4.69 | 39.02 ± 5.91 | 9.85 ± 0.90 |
| **PS1** | 85.49 ± 12.94 | 61.84 ± 8.28 | 199.38 ± 15.04 | 25.90 ± 4.55 | 36.08 ± 3.51 | 13.46 ± 1.29 |
| **PS2** | 90.37 ± 9.47 | 105.29 ± 13.50 | 173.00 ± 15.40 | 40.67 ± 6.06 | 36.76 ± 5.28 | 10.09 ± 1.77 |
| **PS3** | 109.02 ± 10.74 | 117.02 ± 10.86 | 288.30 ± 48.78 | 64.63 ± 7.14 | 47.89 ± 7.70 | 16.60 ± 2.06 |
| **PS4** | 95.50 ± 7.42 | 110.36 ± 12.14 | 230.50 ± 38.80 | 46.09 ± 8.08 | 49.94 ± 7.12 | 12.20 ± 1.03 |
| **PS5** | 125.49 ± 9.44 | 114.40 ± 9.61 | 259.20 ± 30.74 | 56.17 ± 3.53 | 36.52 ± 4.81 | 13.90 ± 2.13 |
| **PS6** | 124.94 ± 18.49 | 101.81 ± 6.74 | 241.40 ± 42.88 | 47.70 ± 5.16 | 44.65 ± 7.50 | 12.20 ± 1.98 |
| **PS7** | 106.58 ± 7.02 | 116.19 ± 15.48 | 244.10 ± 19.52 | 41.16 ± 4.12 | 40.61 ± 5.22 | 13.00 ± 0.86 |
| **PS8** | 123.24 ± 21.73 | 110.03 ± 16.50 | 252.70 ± 24.15 | 55.46 ± 9.28 | 38.11 ± 4.79 | 12.50 ± 0.96 |
| **PS9** | 102.73 ± 7.50 | 107.50 ± 7.09 | 202.30 ± 32.75 | 40.43 ± 6.60 | 47.73 ± 6.27 | 16.80 ± 2.16 |
| **PVC1** | 356.25 ± 27.91 | 199.13 ± 32.07 | 94.51 ± 8.34 | 308.46 ± 46.16 | 79.46 ± 6.96 | 53.32 ± 9.58 |
| **PVC2** | 388.86 ± 55.30 | 219.16 ± 18.88 | 123.42 ± 15.90 | 271.84 ± 26.86 | 72.01 ± 6.36 | 48.66 ± 4.71 |
| **PVC3** | 399.72 ± 31.17 | 265.96 ± 17.84 | 136.05 ± 9.98 | 386.46 ± 55.06 | 72.11 ± 9.90 | 36.64 ± 5.09 |
| **PVC4** | 427.56 ± 65.35 | 214.01 ± 29.95 | 134.02 ± 13.64 | 353.72 ± 58.31 | 84.19 ± 12.96 | 5.77 ± 1.02 |
| **PVC5** | 476.67 ± 51.71 | 296.67 ± 45.95 | 106.84 ± 9.65 | 389.80 ± 46.57 | 85.34 ± 9.31 | 55.10 ± 6.20 |
| **PVC6** | 395.00 ± 26.09 | 301.63 ± 29.91 | 154.75 ± 27.26 | 375.39 ± 29.96 | 98.58 ± 17.24 | 47.57 ± 3.16 |
| **PVC7** | 500.50 ± 83.39 | 240.59 ± 39.21 | 121.83 ± 7.96 | 381.40 ± 42.39 | 66.66 ± 9.80 | 55.82 ± 7.17 |
| **PVC8** | 385.26 ± 23.94 | 261.35 ± 40.90 | 107.23 ± 15.02 | 396.81 ± 61.57 | 95.17 ± 15.58 | 50.07 ± 7.18 |
| **PVC9** | 400.83 ± 40.11 | 227.08 ± 32.48 | 136.50 ± 14.47 | 290.22 ± 22.33 | 71.57 ± 6.00 | 46.89 ± 5.67 |
| **Sample** | **Content (Mean ± SD, µg/kg of dried sample)** | | | | |  |
|  | **Benzo[a]pyrene** | **Indeno[1,2,3-cd]pyrene** | **Dibenz[a,h]anthracene** | **Benzo[g,h,i]perylene** | **Total PAHs** |  |
| **W1** | 1.91 ± 0.22 | 1.50 ± 0.27 | 1.20 ± 0.20 | 1.89 ± 0.25 | 539.50 ± 47.15 |  |
| **W2** | 2.50 ± 0.30 | 1.99 ± 0.17 | 1.20 ± 0.13 | 2.21 ± 0.22 | 491.55 ± 64.55 |  |
| **W3** | 4.38 ± 0.64 | 3.10 ± 0.38 | 1.88 ± 0.21 | 2.59 ± 0.19 | 500.98 ± 56.84 |  |
| **W4** | 3.13 ± 0.27 | 2.57 ± 0.16 | 4.36 ± 0.49 | 2.54 ± 0.36 | 519.23 ± 72.37 |  |
| **W5** | 3.29 ± 0.25 | 2.22 ± 0.30 | 4.97 ± 0.58 | 2.12 ± 0.37 | 641.05 ± 103.24 |  |
| **W6** | 3.17 ± 0.30 | 1.98 ± 0.25 | 4.74 ± 0.63 | 2.58 ± 0.27 | 526.94 ± 74.09 |  |
| **W7** | 3.05 ± 0.48 | 2.29 ± 0.28 | 1.68 ± 0.30 | 2.44 ± 0.42 | 630.58 ± 68.25 |  |
| **W8** | 3.69 ± 0.65 | 2.09 ± 0.13 | 1.39 ± 0.13 | 2.24 ± 0.20 | 613.95 ± 66.90 |  |
| **W9** | 3.56 ± 0.24 | 2.48 ± 0.18 | 1.66 ± 0.27 | 2.37 ± 0.18 | 533.29 ± 54.22 |  |
| **PE1** | 4.68 ± 0.41 | 7.43 ± 1.28 | 5.20 ± 0.57 | 3.73 ± 0.58 | 3442.35 ± 369.97 |  |
| **PE2** | 6.08 ± 0.47 | 14.26 ± 2.38 | 10.63 ± 1.89 | 3.81 ± 0.43 | 3189.45 ± 505.35 |  |
| **PE3** | 6.46 ± 0.94 | 16.19 ± 1.16 | 12.91 ± 2.15 | 4.01 ± 0.56 | 2479.24 ± 352.15 |  |
| **PE4** | 6.81 ± 0.62 | 7.14 ± 0.95 | 9.64 ± 1.33 | 5.45 ± 0.79 | 3673.19 ± 476.32 |  |
| **PE5** | 5.39 ± 0.43 | 14.56 ± 2.32 | 8.97 ± 0.62 | 3.92 ± 0.40 | 3343.70 ± 475.17 |  |
| **PE6** | 5.52 ± 0.38 | 14.70 ± 1.64 | 11.69 ± 1.01 | 4.61 ± 0.43 | 3304.58 ± 389.74 |  |
| **PE7** | 6.24 ± 0.61 | 13.78 ± 2.14 | 10.14 ± 0.98 | 4.28 ± 0.59 | 3530.92 ± 421.45 |  |
| **PE8** | 5.99 ± 0.79 | 11.99 ± 2.13 | 12.43 ± 1.77 | 4.85 ± 0.49 | 3248.91 ± 450.89 |  |
| **PE9** | 5.38 ± 0.46 | 11.93 ± 2.01 | 1.54 ± 0.19 | 4.73 ± 0.61 | 3288.01 ± 462.06 |  |
| **PS1** | 72.01 ± 12.83 | 21.91 ± 3.66 | 13.04 ± 1.00 | 21.21 ± 2.16 | 4225.36 ± 417.97 |  |
| **PS2** | 77.85 ± 10.50 | 28.12 ± 3.64 | 13.20 ± 0.99 | 51.92 ± 6.48 | 4083.58 ± 649.56 |  |
| **PS3** | 60.33 ± 8.27 | 30.15 ± 2.03 | 27.00 ± 4.75 | 86.02 ± 8.30 | 3775.29 ± 470.14 |  |
| **PS4** | 63.30 ± 8.36 | 26.90 ± 3.28 | 20.60 ± 2.70 | 61.39 ± 8.67 | 4622.12 ± 531.54 |  |
| **PS5** | 80.80 ± 14.05 | 26.30 ± 3.97 | 17.50 ± 1.80 | 61.02 ± 6.56 | 4718.27 ± 668.06 |  |
| **PS6** | 76.60 ± 8.09 | 31.80 ± 3.34 | 17.20 ± 1.23 | 61.25 ± 6.21 | 4877.71 ± 635.00 |  |
| **PS7** | 72.40 ± 6.48 | 26.60 ± 3.59 | 15.80 ± 2.56 | 62.96 ± 4.30 | 4519.16 ± 481.05 |  |
| **PS8** | 78.40 ± 10.06 | 27.80 ± 4.29 | 17.40 ± 2.18 | 64.32 ± 5.81 | 4793.18 ± 568.20 |  |
| **PS9** | 63.50 ± 4.08 | 25.30 ± 4.16 | 21.10 ± 3.11 | 49.16 ± 5.99 | 4617.50 ± 468.66 |  |
| **PVC1** | 42.11 ± 3.60 | 63.17 ± 9.30 | 12.93 ± 0.92 | 28.38 ± 3.28 | 3408.62 ± 370.64 |  |
| **PVC2** | 47.03 ± 5.28 | 67.98 ± 4.31 | 13.27 ± 2.03 | 38.60 ± 3.30 | 3448.22 ± 354.94 |  |
| **PVC3** | 49.31 ± 4.28 | 75.30 ± 11.09 | 27.13 ± 3.28 | 96.62 ± 12.44 | 3706.71 ± 435.22 |  |
| **PVC4** | 56.36 ± 8.38 | 89.11 ± 11.94 | 19.54 ± 2.34 | 57.91 ± 5.15 | 4181.39 ± 521.84 |  |
| **PVC5** | 44.71 ± 5.32 | 87.82 ± 6.52 | 23.07 ± 4.12 | 70.19 ± 7.02 | 4112.97 ± 439.34 |  |
| **PVC6** | 53.08 ± 8.47 | 88.83 ± 15.25 | 18.53 ± 1.52 | 56.52 ± 9.11 | 3773.60 ± 461.61 |  |
| **PVC7** | 44.65 ± 4.36 | 80.59 ± 11.99 | 19.72 ± 2.55 | 67.22 ± 10.60 | 4058.13 ± 489.56 |  |
| **PVC8** | 42.03 ± 5.61 | 69.84 ± 8.33 | 22.84 ± 2.81 | 63.54 ± 6.05 | 4002.02 ± 584.34 |  |
| **PVC9** | 46.97 ± 4.77 | 70.03 ± 8.06 | 22.30 ± 1.74 | 55.55 ± 9.63 | 4029.82 ± 456.61 |  |

Table S4. Benjamini–Hochberg adjusted p-values from post-hoc pairwise comparisons of total PAHs in shiitake mushrooms smoked with different fuel types (W, PE, PS, PVC). All plastic-adulterated groups differed significantly from the wood control (W vs PE, W vs PS, W vs PVC, adjusted p < 0.001). Additional significant differences were observed between PE and PS (adjusted p < 0.001) and between PE and PVC (adjusted p = 0.0024).

| Comparison | BH-adjusted p-value |
| --- | --- |
| W vs PE | 0.000618442 |
| W vs PS | 0.000618442 |
| W vs PVC | 0.000618442 |
| PE vs PS | 0.000618442 |
| PE vs PVC | 0.002397228 |
| PS vs PVC | 0.003568628 |
